# Keeping toxigenic *Aspergillus* section *Flavi* and aflatoxin contamination at bay by deploying atoxigenic-based biocontrol products during production of groundnut and maize in Mozambique

**DOI:** 10.3389/fmicb.2024.1501924

**Published:** 2024-11-20

**Authors:** Joao Augusto, Joseph Atehnkeng, Alejandro Ortega-Beltran, Peter J. Cotty, Ranajit Bandyopadhyay

**Affiliations:** ^1^Plant Health and Mycotoxin Unit, International Institute of Tropical Agriculture (IITA), Nampula, Mozambique; ^2^Pathology and Mycotoxin Unit, IITA, Ibadan, Nigeria; ^3^United States Department of Agriculture - Agricultural Research Service (USDA-ARS), Tucson, AZ, United States; ^4^College of Food Science and Engineering, Ocean University of China, Qingdao, China

**Keywords:** *Arachis hypogaea*, *Zea mays*, *Aspergillus* section *Flavi*, Aflasafe, bioprotectant, atoxigenic fungi

## Abstract

Aflatoxins, produced by aflatoxigenic *Aspergillus* section *Flavi* fungi, commonly occur in groundnut and maize grown in Mozambique and have long been associated with high prevalence of liver cancer, stunting, and restricted access to lucrative international markets. Effective aflatoxin control options in the country are limited and not adequately explored. Biocontrol products based on atoxigenic strains of *A. flavus* provide viable aflatoxin mitigation measures but require development for Mozambique. Four hundred and sixty-eight (468) and 558 groundnut and maize farmers, respectively, voluntarily evaluated the effectiveness of two biocontrol products (Aflasafe MWMZ01 and Aflasafe MZ02), each containing as active ingredients four distinct atoxigenic isolates of *A. flavus* belonging to native vegetative compatibility groups (VCGs), at preventing aflatoxin contamination and displacement of aflatoxigenic fungi for 2 years in various agro-ecologies. Most groundnut and maize treated with the biocontrol products were below maximum levels for food in the European Union (EU; 85%; *p* < 0.01) and the United States (US; 99%; *p* < 0.01). In contrast, most non-treated maize and groundnut (ranging from 38 to 70%; *p* = 0.05) were above the EU and US maximum allowable levels for food. Aflatoxin reductions ranged from 78 to 98% (*p* < 0.01) in treated groundnut, and from 61 to 93% (*p* < 0.01) in treated maize. Toxigenic fungi were almost completely displaced from soils and crops by the applied atoxigenic active ingredients. This study revealed that the atoxigenic based biocontrol technology is effective in Mozambique at displacing aflatoxigenic fungi and reducing aflatoxin accumulation in both groundnut and maize but a combination with other management tools is encouraged for better retention of crop quality along the value chain.

## Introduction

Opportunistic fungal infection before and after harvest by aflatoxigenic *Aspergillus* section *Flavi* members and subsequent aflatoxin accumulation in groundnut (*Arachis hypogaea*) and maize (*Zea mays*) are some of the most important challenges faced by farmers and consumers in Mozambique and elsewhere. Several studies had shown association between high prevalence of liver cancer in Mozambique, which ranked among the world’s highest, with aflatoxin intake in prepared foods (Sineque et al., 2019; International Agency for Research on Cancer, 2002). Elsewhere, within the sub-Saharan Africa (SSA) region, exposure to aflatoxins has been associated with stunting, particularly in children under 5 years of age (Rasheed et al., 2021), although in the context of Mozambique these dose response relationships between aflatoxin exposure *in utero* or early infancy and growth impairment have not been clearly established. However, stunting levels are particularly high in prominent maize and groundnut growing regions of Mozambique (Castigo and Salvucci, 2014).

The Rapid Alert System for Food and Feed (RASFF) reported several notifications of Mozambican groundnut products exported to the European Union (EU) that exceeded maximum allowable levels within that market block (RASFF, 2009, 2010). As a result, marketing companies ceased acquiring groundnut in Mozambique for export to the EU and farmers lost access to lucrative markets and important means of survival.

Maize and groundnut, major contributors to dietary intake and income, are prone to aflatoxin contamination in Mozambique (Meijer et al., 2021; Cambaza et al., 2018), particularly when crops are exposed to drought and high temperatures (Cole et al., 1984; Payne, 1998). Maize is notably cultivated in the mid and highland agro-ecological zones (AEZs) (i.e., R4, R7, and R10) while groundnut is commonly grown from the low to midland AEZs (i.e., R7 and R8) in northern and central Mozambique (IIAM, 2006). The lowland AEZ R8 has a semi-arid humid and semi-arid dry climate characterized by lixisols, leptosols, and arenosols and annual rainfall between 800 and 1,200 mm. The midland AEZ R4 is characterized by a semi-arid humid and sub-humid climate with predominately ferralsols and luvisols and annual rainfall between 1,000 and 1,200 mm. The mid to highland AEZ R7 has a semi-arid humid and sub-humid climate typically with ferralsols, luvisols, and acrisols and annual rainfall between 1,000 and 1,200 mm. AEZ R10 is a high-altitude tropical climate with predominantly reddish and slender clay loam soils and annual rainfall between 1,400 and 1,800 mm. The characteristic dry spells, when associated with cultivation of aflatoxin-prone crops, in AEZ R8, and to some extent AEZ R7, may prompt aflatoxin accumulation (Augusto et al., 2014; Cole et al., 1984; Payne, 1998).

Across the globe, aflatoxigenic *Aspergillus* section *Flavi* has been isolated and characterized and aflatoxin content quantified in both maize and groundnut (Klich, 2007; Probst et al., 2011). Individually, species and morphotypes within species of *Aspergillus* section *Flavi* have varying abilities to produce aflatoxins and most do not appear to have specialization or preference to a particular host (St. Leger et al., 2000). In Mozambique, the *A. flavus* L-morphotype and *A. parasiticus* were the most frequently isolated fungi, but fungi with S morphology and *A. tamarii* were also isolated from groundnut and maize soil samples at harvest. Groundnut had the highest aflatoxin levels (up to 5,674 parts per billion; ppb) compared to maize (up to 687 ppb) (Augusto et al., 2014). In another study, *A. flavus* L-morphotype had the highest frequency of isolation (98%) followed by *A. parasiticus* (1.5%) and fungi with S morphology (0.2%) from maize collected after harvest but there were no detectable aflatoxin levels in the examined maize (Probst et al., 2014). However, a previous study (Warth et al., 2012) had found high aflatoxin content in maize (69.9 ppb) compared to groundnut (3.4 ppb). Nonetheless, in these last two studies there was no indication of specific AEZs or locations from where the samples were collected.

Aflatoxin control measures in crops in the field or after harvest are primarily directed at controlling the aflatoxigenic fungi or restricting aflatoxin production. Maize and groundnut germplasm with resistance to aflatoxin has been reported (Holbrook et al., 1997; Menkir et al., 2006). Candidate target proteins encoding resistance genes associated with aflatoxin accumulation in both crops have been identified (Wang et al., 2016; Dhakal et al., 2017), but specific mechanisms of resistance need to be further identified and elucidated (Soni et al., 2020) and in many cases molecular resistance mechanisms do not necessarily translate into effective field resistance. Cultural practices may reduce crop aflatoxin content by reducing plant stress through use of optimal plant density and planting time, adapted varieties, weed control, fertilization, liming, and crop irrigation. Insect management and harvesting at physiological maturity may also limit *Aspergillus* infection and subsequent aflatoxin production (Klich, 2007). The ‘dry chain’ approach is also necessary to keep grain moisture low during storage (Bradford et al., 2020).

Although aflatoxins cannot be degraded by normal cooking, alkaline cooking (nixtamalization) processes reduce maize aflatoxin content (Méndez-Albores et al., 2004a), but this detoxification is reversible by acidification (Méndez-Albores et al., 2004b) which can occur during digestion. Roasting, irradiation, and fumigation can achieve some degree of aflatoxin reduction (Markov et al., 2015; Emadi et al., 2022). Adsorbants, ammoniation, and color-sorting (Park et al., 1988) are commercially used to lower crop aflatoxin content. Products subjected to these post-harvest aflatoxin detoxification/removal practices may also extract essential nutrients, not achieve acceptable levels of aflatoxin reduction, cause wasteful discarding of crop components, or unsafe for consumption (Gómez-Salazar et al., 2023). Available practices individually or in combination are complex for most farmers to implement and are not always possible. Even if such practices are implemented, fungal infection and aflatoxin accumulation beyond acceptable levels may still occur under highly conducive environmental events (Cotty and Jaime-Garcia, 2007).

Biocontrol is mostly based on competitive exclusion of aflatoxigenic fungi by non-aflatoxigenic (atoxigenic) isolates of *A. flavus* to limit aflatoxin accumulation in crops but not necessarily fungal inoculum (Cotty, 1994; Mehl et al., 2012). Reasons for atoxigenicity include SNPs, deletions, and/or insertions in genes of the aflatoxin biosynthetic pathway (Adhikari et al., 2016). There is also evidence that the inhibition of aflatoxin production is thigmoregulated and aflatoxin biosynthesis genes of aflatoxigenic fungi are down regulated when in contact with antagonistic atoxigenic isolates (Rao et al., 2020). Early consistent findings of competitive displacement of aflatoxigenic fungi by atoxigenic isolates of *A. flavus* and aflatoxin reduction were reported in cotton (Cotty, 1990, 1994), groundnut (Dorner et al., 1992), and maize (Brown et al., 1991). Atoxigenic *A. flavus*-based biocontrol technologies have since been developed and used in cotton (Cotty et al., 2007), groundnut (Dorner and Lamb, 2006), maize (Abbas et al., 2006), pistachio (Doster et al., 2014), almond and fig (Ortega-Beltran et al., 2019). In Africa, aflatoxin biocontrol innovations, with “Aflasafe” trademark, based on formulations containing four atoxigenic isolates of *A. flavus* native to target countries have been widely tested and used in both maize and groundnut (Bandyopadhyay et al., 2022; Agbetiamegh et al., 2020; Senghor et al., 2021; Mahuku et al., 2023). These country- or within region-specific meticulously selected and highly competitive, adapted atoxigenic isolates of *A. flavus* offer short- and long-term solutions to aflatoxin contamination from the field to storage.

The current study aimed to assessing the efficacy of two bioprotectants containing atoxigenic isolates of *A. flavus* belonging to atoxigenic vegetative compatibility groups (VCGs) native to Mozambique on displacement of aflatoxigenic fungi and aflatoxin reduction in maize and groundnut grown across several AEZs in Mozambique. The results from this study provide the first evidence of utility of aflatoxin biocontrol in Southern Africa, useful information on *A. flavus* population dynamics, and aflatoxin management tools effective in Mozambique, and of potential value in other regions with similar agro-ecologies.

## Materials and methods

### Locations of study

Volunteer participatory farmers were selected from agro-ecological zones (AEZs) R4 (Manica province), R7 (Nampula, Niassa, and Zambezia provinces), R8 (Nampula province), and R10 (Zambezia and Tete provinces) in Mozambique (IIAM, 2006) to host farmer field trials to evaluate effectiveness of aflatoxin biocontrol products against aflatoxigenic fungi and aflatoxin accumulation. The trials were conducted from December to May during 2016/2017 and 2017/2018 cropping seasons. Each farmer was selected based on willingness and ability to participate in the trials and possession of about one hectare (ha) field to apply the treatments. To avoid cross-contamination of the applied products after the sporulation of the active ingredients on the formulation substrate (see below for method of formulation), each farmer received one treatment and separation among treated and non-treated fields was about 400 meters. There were 66, 174, 210, and 18 groundnut farmers for AEZs R4, R7, R8, and R10, respectively, during the two cropping seasons. Similarly, there were 84, 126, 102, and 246 maize farmers for AEZs R4, R7, R8, and R10, respectively. The number of farmers per AEZ was proportional to the area cultivated for each crop in the AEZ.

### The biocontrol products and their formulations

Two aflatoxin bioprotectants, namely Aflasafe MWMZ01 and Aflasafe MZ02, were evaluated. Each bioprotectant contained as active ingredient four different, highly competitive atoxigenic isolates of *A. flavus* L-morphotype belonging to atoxigenic VCGs widely distributed across Mozambique (active ingredients – 0.0005%). The atoxigenic VCGs were detected through VCG analysis as previously described (Atehnkeng et al., 2016; data not presented). Aflasafe MWMZ01 contained isolates GP5G-8, GP1H-12, MZM594-1, and MZM029-7, while Aflasafe MZ02 contained isolates GP5G-8, MZG071-6, MZM028-5, and MZM250-8. The atoxigenic fungi were selected from a pool of about 3,000 isolates obtained from maize and groundnut grown across Mozambique from 2013 to 2015.

For each Aflasafe bioprotectant, a suspension containing equal proportions of spores from the four atoxigenic active ingredients (10 mL/kg) was coated on roasted, sterile sorghum grains with the aid of a polymer (1.5 mL/kg) and a blue food dye (2 mL/kg) in distilled water (10.5 mL/kg) using an industrial process (Bandyopadhyay et al., 2016). The products were manufactured in IITA premises in Dar es Salaam, Tanzania.

### Experimental fields and treatments

In a set of three 1-ha fields (one field per farmer) for groundnut or maize, each field was treated with either Aflasafe MZMW01, Aflasafe MZ02, or left non-treated as negative control. The three fields in a set were separated by at least 400 m. There were 22, 58, 70, and 6 treatment sets (replications) for groundnut fields in AEZs R4, R7, R8, and R10 respectively, and 28, 42, 34, and 82 treatment sets (replications) for maize fields in AEZs R4, R7, R8, and R10, respectively, during the 2016/2017 and 2017/2018 cropping seasons. The field experiments, for each crop and AEZ, were in a randomized complete block design.

Fields were planted with groundnut and maize farmers’ preferred varieties in December and harvested in April (groundnut) and May (maize). Both crops were rainfed and no fungicides were applied. Groundnut was planted at seeding rate of 77 kg/ha (one seed/hole) and maize was seeded at 53 kg/ha (three seeds/hole). Biocontrol products were uniformly applied by the farmers at a rate of 10 kg/ha by hand about 30 days after groundnut planting when canopy was expanding, or 2 to 3 weeks before maize flowering. Farmers performed all field maintenance and agronomic practices according to recommendations of the local agricultural extension norms.

### Soil and crop sampling

In each groundnut or maize field at planting (before biocontrol treatment) and at harvesting (after biocontrol treatment), five soil subsamples were randomly taken with a probe at depth of ≤5 cm from each of four marked quadrants, and the 20 subsamples combined in Ziploc bags to a total weigh of approximately 500 g and transported to the IITA Plant Health and Mycotoxin Laboratory in Nampula, Mozambique. The soil samples were then oven-dried at 40°C for 5 days and stored at room temperature (~25°C) (Cardwell and Cotty, 2002) for fungal analysis. At harvest, maize cobs or unshelled groundnut were randomly taken from four quadrants and pooled to a total of 28 maize cobs or 1 kg groundnut in each field and transported to the laboratory in Nampula. During storage, approximately 3 months after harvest, 15 maize cobs and 500 g shelled groundnut from biocontrol treated and non-treated fields were uniformly collected from farmers’ stores and transported to the laboratory in Nampula. De-husked maize cobs and unshelled groundnut samples at harvest were air-dried to maximum moisture content of 13 and 9%, respectively. Maize and groundnut were then shelled and ground to fine particles (< 20 μm) using a blender (Waring Commercial, Torrington, CT). The blender container and lid were washed with soap, 5% bleach, rinsed with 100% ethanol, and let to air dry before the next sample was processed to avoid cross-contamination among samples. The ground samples were stored at 5°C until use for fungal and aflatoxin analyses.

### Aflatoxin analysis

Total aflatoxins in maize and groundnut samples were quantified in crops at harvest using the Reveal® Q+ kit (Reveal Q+ for aflatoxin with AccuScan testing system, Neogen Corporation, Lansing, MI) following the manufacturer’s instructions. For each sample, a 20 g sub-sample was combined with 100 mL 65% ethanol and blended for 1 min. The mixture was passed through Whatman No. 1 filter paper (Whatman Intl. Ltd., Maidstone, England) and the filtrate (100 μL) was added to a diluent (500 μL) and mixed by inverting tubes 5 times. The mixture (100 μL) was transferred to a new tube and an aflatoxin strip was placed into the tube and kept for 6 min before reading in the AccuScan testing system. Reveal® Q+ kit quantifies total aflatoxins in the range of 2–150 ppb. Values above the upper limit were diluted and read again to bring the quantification inside the range.

### *Aspergillus* section *Flavi* mycoflora analysis for soil and crop samples

*Aspergillus* section *Flavi* members were isolated from soil before and after biocontrol treatment (at harvest), and maize grain and groundnut kernels at harvest and after 3-months of storage with a dilution plate technique on modified rose Bengal agar (MRBA) (Probst et al., 2011). Briefly, 1 g homogenized soil, ground maize grain, or ground groundnut kernels was suspended in 10 mL sterile distilled water in a 40 mL glass vial (Thermo Fisher Scientific, Rockwood, TN), mixed using an analog vortex mixer (Thomas Scientific, Swedesboro, NJ) for 2 min, and appropriate dilutions plated on MRBA. Inoculated plates were incubated for 3 days in the dark (31°C). *Aspergillus* colonies from plates with ≤10 colonies were transferred to 5–2 agar [5% V-8 vegetable juice (Campbell Soup Co., Camden, NJ), 2% Bacto-agar (Becton, Dickinson and Company, Sparks, MD), pH 6.0] and incubated (31°C) for 5 days. The isolated *Aspergillus* section *Flavi* members were classified based on colony macroscopic and microscopic (×400 magnification) characteristics (Garber and Cotty, 1997; Cotty and Cardwell, 1999). The *A. flavus* L-morphotype produced characteristically light green colonies and few, large sclerotia (>400 μm, avg. diameter). Fungi producing numerous small sclerotia (<400 μm, average diameter) were classified as fungi with S morphology. There are several species having this morphology but their assignment to appropriate species requires molecular characterization; this was not done in the current study. The *A. parasiticus* members produced their distinguished dark-green colonies while *A. tamarii* colonies were dark brown. The isolation frequency and aflatoxin production of *A. tamarii* were negligible and, therefore, this fungus was excluded from the final count and aflatoxin analysis.

### Toxigenicity of *Aspergillus* section *Flavi* isolates

We analyzed aflatoxin production potential of 3,126 isolates obtained from soil during planting and harvesting of groundnut and maize, grains of groundnut and maize at harvesting and 3 months after storage across all AEZs and treatments during both years. *In vitro* aflatoxin production ability of *A. flavus* L-morphotype, fungi with S morphology, and *A. parasiticus* isolates was determined on undamaged and autoclaved aflatoxin-free maize grains as previously described (Probst and Cotty, 2012). Briefly, 5 g aflatoxin-free maize grains were washed twice with tap water, soaked overnight in 25 mL of tap water to adequately boost grain moisture content to support fungal growth when inoculated. The soaking water was then drained, the grains rinsed twice with tap water, and autoclaved (121°C) for 20 min in 40 mL polystyrene glass vials to sterilize and remove any fungal contaminants. Sterilized grains were individually inoculated with 500 μL spore suspension of each *Aspergillus* isolate containing approximately 10^6^ conidia per vial and incubated for 7 days at 31°C in the dark, including inoculation and incubation of negative control maize vial pairs with 500 μL sterile distilled water. After incubation, 50 mL 70% methanol were added, the maize-methanol mixture was homogenized with a blender (Waring Commercial, Torrington, CT), and aflatoxins were extracted and quantified using the Neogen Accuscan testing system with the Reveal® Q+ kit as described above. *Aspergillus flavus* L-morphotype isolates producing no detectable (i.e., <2 ppb) aflatoxins were termed atoxigenic, while *A. flavus* L-morphotype, fungi with S morphology, and *A. parasiticus* with quantifiable aflatoxins were designated toxigenic. Then, the percentages of atoxigenic isolates were determined for each treatment sample in all AEZs and both crops.

### Statistical analysis

Aflatoxins in both maize and groundnut at harvest across AEZs were grouped into 3 categories according to the aflatoxin accumulation levels to determine the percentage of crops associated with those categories. These were ≤4 (ppb; EU aflatoxin maximum legal limit for food consumption), ≤20 ppb (US aflatoxin maximum legal limit for food consumption), and >20 ppb (CODEX unacceptable guidance levels for food consumption). Because of highly skewed aflatoxin data of the control treatments, groundnut and maize aflatoxin data across AEZs in the biocontrol experiments were log-transformed [Log (aflatoxin concentration + 1)] before statistical analysis to normalize the variance. All data were subjected to statistical analysis using the mixed procedure (PROC MIXED) of SAS v9.4 (SAS Institute, Cary, NC), where year (qualitative), AEZ (qualitative), crop (qualitative), aflatoxin category (quantitative), and biocontrol treatments (qualitative) were considered fixed-effects parameters, while percentage of crop fields associated with aflatoxin categories, log aflatoxin data, number of atoxigenic and toxigenic isolates, isolation frequency of *Aspergillus* section *Flavi* isolates in the soil, groundnut kernels, and maize grain were modeled as covariance or random-effects parameters. The CLASS statement was invoked for year, AEZ, crop, aflatoxin category, and biocontrol treatments as classification variables. The MODEL statement specified the response variables percentage of crop fields, log aflatoxin data, number of atoxigenic and toxigenic isolates, isolation frequency of *Aspergillus* section *Flavi* isolates in the soil, groundnut kernels, and maize grain. Then, the classification variables and their interactions were listed after the equal (=) sign (in the MODEL statement). The RANDOM statement was invoked for aflatoxin category ranges and all its interactions with the classification variables. The RUN statement completed the specifications. The year, AEZ, crop, aflatoxin category, and biocontrol treatment effects were assessed by using the generalized least-square means.

## Results

### Interactions between AEZ, crops, *Aspergillus* and aflatoxin

The analysis of variance output showed no significant interaction year × frequency of isolation of *Aspergillus* section *Flavi* fungi, year × aflatoxin content in both groundnut and maize. Therefore, the data of both years were combined. The outputs of “Type 3 Tests of Fixed Effects” for the response variables showed that the four-way interaction AEZ × crop × aflatoxin category × biocontrol treatment was significant (*p* = 0.028) for the percent of crops at harvest associated with aflatoxin categories. The three-way interaction AEZ × crop × biocontrol treatment was significant for the Log aflatoxin content (*p* < 0.01), percentages of *Aspergillus* section *Flavi* isolates in the soil from groundnut (*p* < 0.01) and maize (*p* < 0.01) fields at harvest after biocontrol treatments, percentages of *Aspergillus* section *Flavi* isolates from groundnut kernels (*p* < 0.01) and maize grain (*p* = 0.034) at harvest, and percentages of *Aspergillus* section *Flavi* isolates from groundnut kernels (*p* = 0.024) and maize grain (*p* = 0.040) during storage (3 months after harvest).

### Field efficacy of Aflasafe MWMZ01 and Aflasafe MZ02 in groundnut and maize

**Figure 1:**
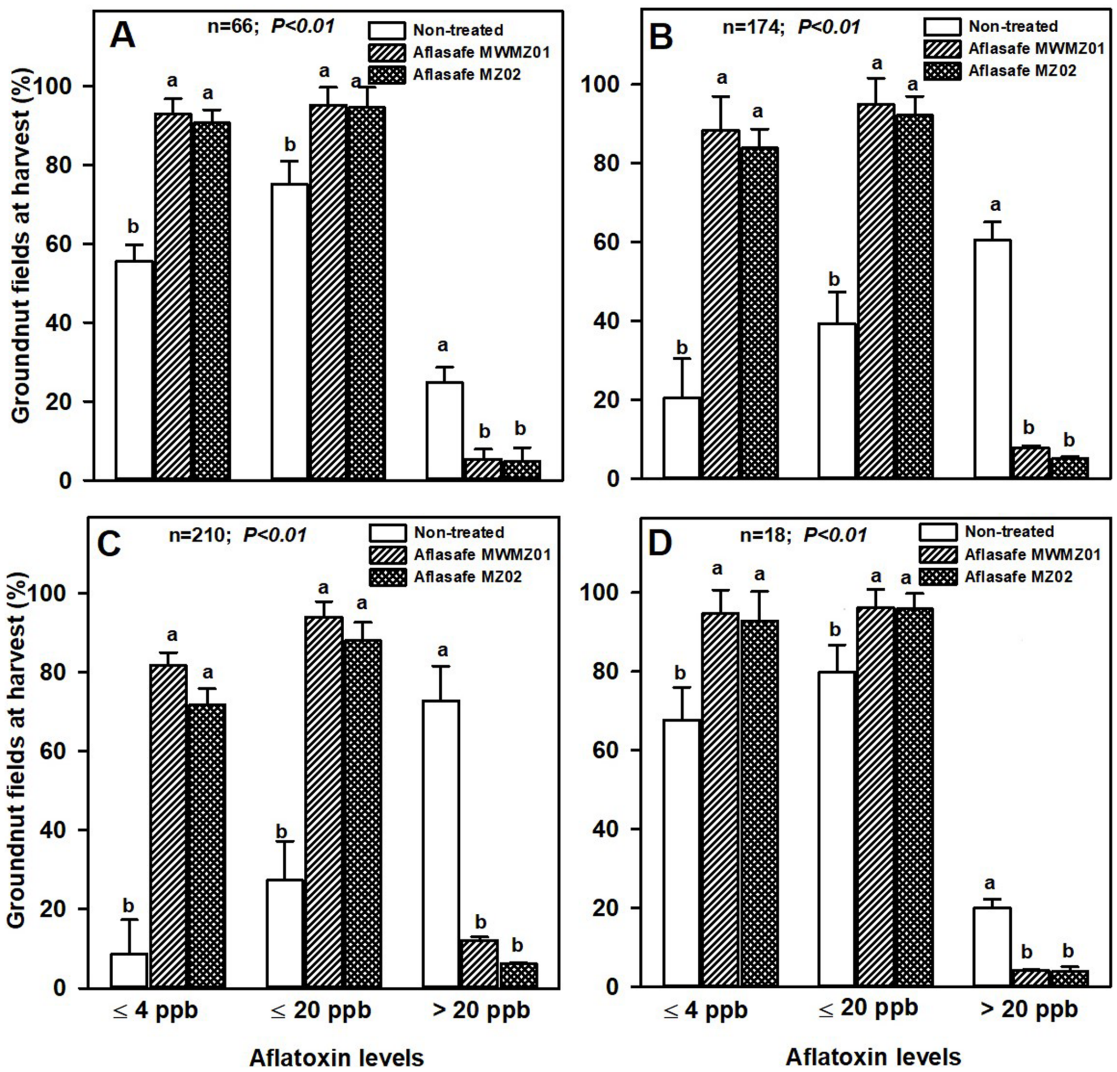
Aflatoxin levels at harvest for groundnut kernels from non-treated fields, and from fields treated with either Aflasafe MWMZ01 or Aflasafe MZ02 biocontrol products in agro-ecological zones R4 **(A)**, R7 **(B)**, R8 **(C)**, and R10 **(D)**. Bar heights are means for percentage groundnut crops, and bars in each aflatoxin category level (≤ 4 ppb, ≤ 20 ppb, or > 20 ppb) with different letters significantly (*p* < 0.01) different according to least significant difference (LSD) test.

About 93 and 96% of the treated groundnut had aflatoxin levels ≤4 ppb and ≤20 ppb, respectively, in AEZs R4 and R10. Comparatively, only about 60 and 78% of non-treated groundnut had aflatoxin levels ≤4 ppb and ≤ 20 ppb, respectively (Figure 1). In the AEZs R7 and R8, where aflatoxin levels were highest in non-treated groundnut with about 67% with >20 ppb, the differences between treated and non-treated groundnut were clearer. On an average, 80 and 93% of the treated groundnut had ≤4 ppb and ≤ 20 ppb, respectively, compared to non-treated groundnut where only 15 and 33% had ≤4 ppb and ≤ 20 ppb, respectively. In each aflatoxin category range (≤ 4 ppb, ≤ 20 ppb, or > 20 ppb), the two biocontrol products were similar in effectiveness across AEZs (Figure 1).

**Figure 2:**
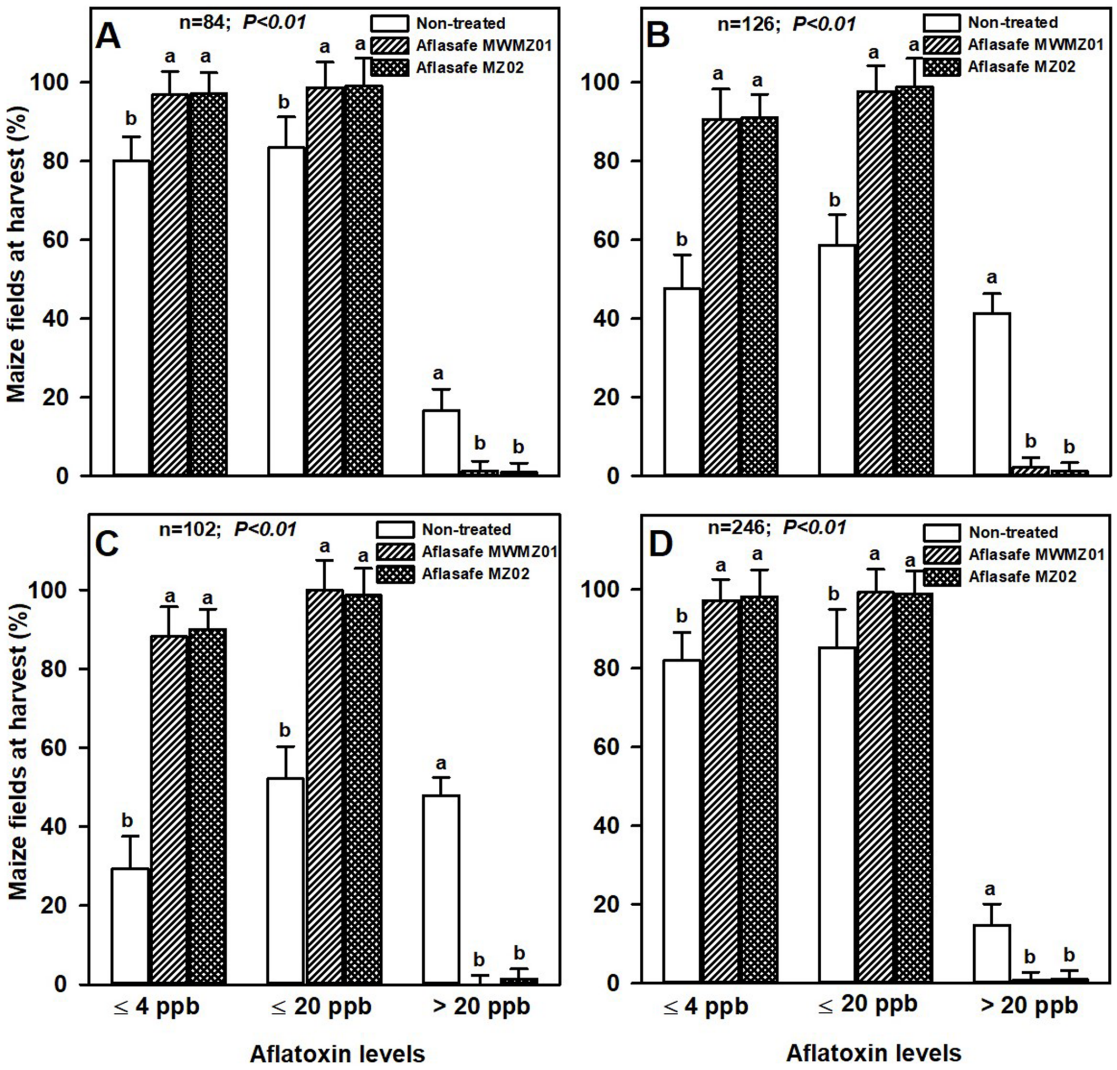
Aflatoxin levels at harvest for maize from non-treated fields, and from fields treated with either Aflasafe MWMZ01 or Aflasafe MZ02 biocontrol products in agro-ecological zones R4 **(A)**, R7 **(B)**, R8 **(C)**, and R10 **(D)**. Bar heights are means for percentage of maize crops, and bars in each aflatoxin category level (≤4 ppb, ≤20 ppb, or >20 ppb) with different letters are significantly (*p* < 0.01) different according to least significant difference (LSD) test.

Maize was relatively less prone to aflatoxin than groundnut. About 45% of non-treated maize had >20 ppb aflatoxin across AEZs R7 and R8 with highest aflatoxin prevalence of all AEZs. About 98 and 99% of the treated maize had ≤4 ppb and ≤ 20 ppb aflatoxin, respectively, across AEZs R4 and R10. For non-treated maize across the same AEZs, 81 and 84% of the maize had ≤4 ppb and ≤ 20 ppb aflatoxin, respectively. The AEZs R7 and R8 in average had 90 and 99% of the treated maize with ≤4 ppb and ≤ 20 ppb aflatoxin, respectively, and in non-treated fields the proportions were lower with 38 and 55% of the maize with ≤4 ppb and ≤ 20 ppb, respectively. Like groundnut, both biocontrol products had comparable effectiveness in maize in each of the AEZ (Figure 2).

**Figure 3:**
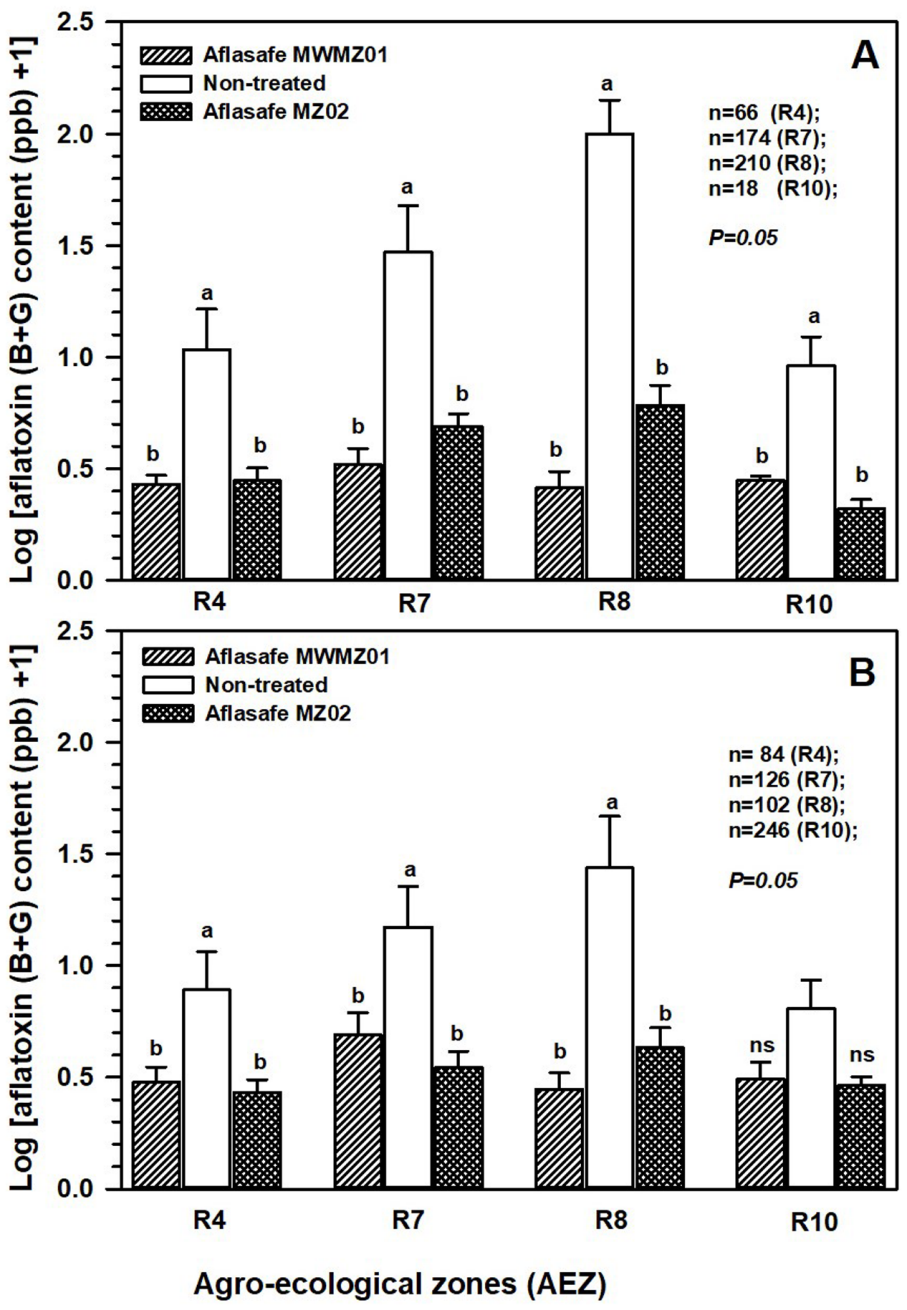
Effectiveness of Aflasafe MWMZ01 and Aflasafe MZ02 biocontrol products on aflatoxin reduction in groundnut **(A)**, and maize **(B)**, compared to crops that received no treatment. Comparisons are presented across agro-ecological zones (AEZ) R4 (midland; Manica province), R7 (mid- to highland; Nampula, Niassa, and Zambezia provinces), R8 (lowland; Nampula province), and R10 (highland; Zambezia and Tete provinces). Bar heights are means for Log (total aflatoxin +1) accumulation, and within each AEZ, treatments with different letters are significantly (*p* = 0.05) different according to least significant difference (LSD) test.

The application of either Aflasafe MWMZ01 or Aflasafe MZ02 had significant (*p* = 0.05) reduction in aflatoxin accumulation compared to groundnut and maize crops grown in non-treated fields in all AEZs. Groundnut had higher aflatoxin prevalence than maize across AEZs but particularly in AEZs R7 and R8 where the two biocontrol products had greater impact in aflatoxin reduction (Figure 3). When examining non-treated crops across AEZs, higher aflatoxin contamination occurred in both maize (*p* = 0.031) and groundnut (*p* = 0.027) in R8, followed by R7, while lesser aflatoxin occurred in R4 and R10.

### Distribution of *Aspergillus* section *Flavi* and displacement of the toxigenic fungi in soils

The community structures in soils before treatment varied among AEZs and crops but there were some trends (Tables 1, 2). In AEZs R4 and R10, the most frequently isolated fungus in groundnut and maize soil at planting and before biocontrol treatment were *A. parasiticus*, followed by *A. flavus* L-morphotype, and fungi with S morphology to a lesser extent (Tables 1, 2). The *A. flavus* L-morphotype, on the other hand, dominated communities prior to treatment in AEZs R7 and R8, in the soils of both crops, followed by *A. parasiticus* and fungi with S morphology (Tables 1, 2). On the other hand, at harvest, after biocontrol application, high percentage isolation of the *A. flavus* L-morphotype were observed from the soils of treated groundnut and maize fields. The *A. flavus* L-morphotype completely dominated the communities at harvest in treated groundnut soil in three AEZs (R4, R7, and R10), and in treated maize soil in the fourth AEZ, R10 (Tables 1, 2). In the rest of the AEZs, the percentage of the *A. flavus* L-morphotype in treated soils at harvest ranged from 84 to 93%. In contrast, in non-treated field soils, both *A. flavus* L-morphotype and *A. parasiticus*, and to a lesser extent fungi with S morphology, were common, at levels similar to those observed at planting, except in R8 where *A. flavus* L-morphotype and fungi with S morphology dominated (Tables 1, 2).

**Table 1:** Composition of *Aspergillus* section *Flavi* communities in soils from non-treated and treated groundnut fields at planting (before biocontrol treatment) and at harvest (after biocontrol treatment) in four agro-ecological zones (AEZ)^Z^.

AEZ	Treatment	*Aspergillus* section Flavi community (%)^X,Y^			
		L	S	P	Atoxigenic
R4 (*n* = 66)	At planting *(before treatment)*				
	Aflasafe MWMZ01	33	11	56	18
	Aflasafe MZ02	39	10	51	18
	Non-treated fields	38	12	50	16
	At harvest *(after treatment)*				
	Aflasafe MWMZ01	100a	0	0b	92
	Aflasafe MZ02	100a	0	0b	90
	Non-treated fields	33b	19	48a	9
R7 (*n* = 174)	At planting *(before treatment)*				
	Aflasafe MWMZ01	62	0	38	9
	Aflasafe MZ02	52	12	36	6
	Non-treated fields	60	9	31	8
	At harvest *(after treatment)*				
	Aflasafe MWMZ01	100a	0	0b	93
	Aflasafe MZ02	100a	0	0b	94
	Non-treated fields	48b	10	42a	6
R8 (*n* = 210)	At planting *(before treatment)*				
	Aflasafe MZMW01	63	11	26	4
	Aflasafe MZ02	60	18	22	7
	Non-treated fields	67	13	20	6
	At harvest *(after treatment)*				
	Aflasafe MWMZ01	89a	0b	11	93
	Aflasafe MZ02	91a	0b	9	95
	Non-treated fields	63b	27a	10	3
R10 (*n* = 18)	At planting *(before treatment)*				
	Aflasafe MWMZ01	39	0	61	24
	Aflasafe MZ02	34	8	58	47
	Non-treated fields	38	12	50	29
	At harvest *(after treatment)*				
	Aflasafe MWMZ01	100a	0	0b	85
	Aflasafe MZ02	100a	0	0b	89
	Non-treated fields	51b	7	42a	24

^Z^ This study was conducted in AEZ R4 (midland; Manica province), R7 (mid- to highland; Nampula, Niassa, and Zambezia provinces), R8 (lowland; Nampula province), and R10 (highland; Zambezia and Tete provinces). ^Y^ L – *A. flavus* L-morphotype, S - fungi with S morphology, and P – *A. parasiticus*. Frequency of isolation of *A. tamarii* were negligible and excluded from the study. Atoxigenic – percentages of all fungi that did not produce detectable quantities of aflatoxins. ^X^ Percentages of each *Aspergillus* section Flavi member in each AEZ followed by different letters are significantly (*p* = 0.05) different according to least significant difference (LSD) test.

**Table 2:** Composition of *Aspergillus* section *Flavi* communities in soils from non-treated and treated maize fields at planting (before biocontrol treatment) and at harvest (after biocontrol treatment) in four agro-ecological zones (AEZ)^Z^.

AEZ	Treatment	*Aspergillus* section Flavi community (%)^X,Y^			
		L	S	P	Atoxigenic
R4 (*n* = 84)	At planting *(before treatment)*				
	Aflasafe MWMZ01	34	17	49	18
	Aflasafe MZ02	33	11	56	19
	Non-treated fields	36	8	56	24
	At harvest *(after treatment)*				
	Aflasafe MWMZ01	91a	0	9b	93
	Aflasafe MZ02	93a	0	7b	93
	Non-treated fields	32b	0	68a	19
R7 (*n* = 126)	At planting *(before treatment)*				
	Aflasafe MWMZ01	67	0	33	8
	Aflasafe MZ02	62	18	20	10
	Non-treated fields	66	10	24	11
	At harvest *(after treatment)*				
	Aflasafe MWMZ01	91a	0	9b	94
	Aflasafe MZ02	84a	10	6b	91
	Non-treated fields	38b	18	44a	11
R8 (*n* = 102)	At planting *(before treatment)*				
	Aflasafe MWMZ01	67	19	14	6
	Aflasafe MZ02	54	20	26	7
	Non-treated fields	60	27	13	8
	At harvest *(after treatment)*				
	Aflasafe MWMZ01	92a	8b	0	94
	Aflasafe MZ02	91a	0b	9	90
	Non-treated fields	52b	48a	0	6
R10 (*n* = 246)	At planting *(before treatment)*				
	Aflasafe MWMZ01	39	10	51	30
	Aflasafe MZ02	43	0	57	29
	Non-treated fields	34	17	49	29
	At harvest *(after treatment)*				
	Aflasafe MWMZ01	100a	0	0b	91
	Aflasafe MZ02	100a	0	0b	96
	Non-treated fields	24b	0	76a	24

^Z^ This study was conducted in AEZ R4 (midland; Manica province), R7 (mid- to highland; Nampula, Niassa, and Zambezia provinces), R8 (lowland; Nampula province), and R10 (highland; Zambezia and Tete provinces). ^Y^ L – *A. flavus* L-morphotype, S – fungi with S morphology, and P – *A. parasiticus*. Frequency of isolation of *A. tamarii* were negligible and excluded from the study. Atoxigenic – percentages of all fungi that did not produce detectable quantities of aflatoxins. ^X^ Percentages of each *Aspergillus* section Flavi member in each AEZ followed by different letters are significantly (*P* = 0.05) different according to least significant difference (LSD) test.

The toxigenic isolates were disproportionally predominant compared to atoxigenic isolates, in all maize and groundnut soils, both prior to treatment and at harvest in non-treated fields (Tables 1, 2). For example, in R7 and R8 groundnut and maize field soils, the percentages of atoxigenic isolates in non-treated fields ranged from only 3–11%. In contrast, in treated field soils at harvest for the same AEZs and crops, these percentages strikingly skewed toward atoxigenic isolates ranging from 90 to 95% (Tables 1, 2). But in less aflatoxin prone AEZs R10 and R4, in groundnut and maize field soils both prior to treatment and at harvest in non-treated fields, had fairly large percentages of atoxigenic isolates (Tables 1, 2).

### Distribution of *Aspergillus* section *Flavi* and displacement of the toxigenic fungi in crops at harvest and after storage

Groundnut kernels and maize grains at harvest from biocontrol treated fields almost exclusively contained *A. flavus* L-morphotype across all AEZs (Table 3). High percentages of atoxigenic fungi were found (from 91 to 97%) in groundnut kernels and maize grains that received treatment. In non-treated fields, *A. flavus* L-morphotype and *A. parasiticus* were frequently isolated from maize grains, while *A. flavus* L-morphotype and fungi with S morphology were the most frequently isolated from groundnut kernels, except for AEZ R10 where *A. flavus* L-morphotype and *A. parasiticus* were commonly isolated. The toxigenic fungi dominated groundnut kernels and maize grains from non-treated fields (Table 3). Likewise, during storage for 3 months after harvest, *A. flavus* L-morphotype continued to be the most frequently isolated fungus in groundnut kernels and maize grains from biocontrol-treated fields across all AEZs (Table 4). The atoxigenic fungi were dominant (66 to 90%) in groundnut kernels and maize grains from treated fields compared to those from non-treated fields, from 3 to 33% (Table 4). Also, *A. flavus* L-morphotype and fungi with S morphology were frequently isolated in groundnut from non-treated fields during storage across AEZs, except for AEZ R10 where *A. flavus* L-morphotype and *A. parasiticus* were commonly isolated, while *A. flavus* L-morphotype followed by *A. parasiticus* (in AEZs R4 and R7) and *vice-versa* (in AEZs R8 and R10) were the most frequently isolated fungi in maize from non-treated fields. The toxigenic fungi were the most dominant in both groundnut kernels and maize grains from non-treated fields (Table 4).

**Table 3:** Composition of *Aspergillus* section *Flavi* communities in groundnut or maize kernels from fields treated with aflatoxin biocontrol products and from non-treated control fields in four agro-ecological zones (AEZ)^Z^ at harvest.

AEZ	Treatment	*Aspergillus* section Flavi community (%)^X,Y^			
		L	S	P	Atoxigenic
R4	Groundnut (*n* = 66)				
	Aflasafe MWMZ01	100a	0b	0	95
	Aflasafe MZ02	94a	6b	0	94
	Non-treated fields	50b	50a	0	17
	Maize (*n* = 84)				
	Aflasafe MWMZ01	100a	0	0b	93
	Aflasafe MZ02	100a	0	0b	93
	Non-treated fields	43b	11	46a	32
R7	Groundnut (*n* = 174)				
	Aflasafe MWMZ01	100a	0b	0	93
	Aflasafe MZ02	92a	8b	0	94
	Non-treated fields	49b	38a	13	7
	Maize (*n* = 126)				
	Aflasafe MWMZ01	100a	0	0b	92
	Aflasafe MZ02	100a	0	0b	94
	Non-treated fields	57b	10	33a	19
R8	Groundnut (*n* = 210)				
	Aflasafe MWMZ01	100a	0b	0	91
	Aflasafe MZ02	100a	0b	0	92
	Non-treated fields	59b	32a	9	5
	Maize (*n* = 102)				
	Aflasafe MWMZ01	100a	0	0b	91
	Aflasafe MZ02	100a	0	0b	91
	Non-treated fields	22b	11	67a	8
R10	Groundnut (*n* = 18)				
	Aflasafe MWMZ01	100a	0	0b	96
	Aflasafe MZ02	100a	0	0b	96
	Non-treated fields	61b	10	29a	20
	Maize (*n* = 246)				
	Aflasafe MWMZ01	100a	0	0b	97
	Aflasafe MZ02	100a	0	0b	97
	Non-treated fields	42b	0	58a	39

^Z^ This study was conducted in AEZ R4 (midland; Manica province), R7 (mid- to highland; Nampula, Niassa, and Zambezia provinces), R8 (lowland; Nampula province), and R10 (highland; Zambezia and Tete provinces). ^Y^ L – A. flavus L-morphotype, S – fungi with S morphology, and P – *A. parasiticus*. Frequency of isolation of *A. tamarii* was negligible and excluded from study. Atoxigenic – percentages of all fungi that did not produce detectable quantities of aflatoxins. ^X^ Percentages of each *Aspergillus* section Flavi member in each AEZ followed by different letters are significantly (*P* = 0.05) different according to least significant difference (LSD) test.

**Table 4:** Composition of *Aspergillus* section *Flavi* communities in groundnut or maize kernels from fields treated with aflatoxin biocontrol products and from non-treated control fields in four agro-ecological zones (AEZ)^Z^ after harvest followed by storage for 3-months.

AEZ	Treatment	*Aspergillus* section Flavi community (%)^X,Y^			
		L	S	P	Atoxigenic
R4	Groundnut (*n* = 66)				
	Aflasafe MWMZ01	88a	10b	2	83
	Aflasafe MZ02	81a	9b	10	66
	Non-treated fields	57b	40a	3	10
	Maize (*n* = 84)				
	Aflasafe MWMZ01	89a	0	11b	80
	Aflasafe MZ02	96a	1	3b	75
	Non-treated fields	51b	0	49a	25
R7	Groundnut (*n* = 174)				
	Aflasafe MWMZ01	80a	8b	12	81
	Aflasafe MZ02	87a	10b	3	80
	Non-treated fields	32b	57a	11	3
	Maize (*n* = 126)				
	Aflasafe MWMZ01	90a	0	10b	82
	Aflasafe MZ02	96a	0	4b	84
	Non-treated fields	47b	9	44a	20
R8	Groundnut (*n* = 210)				
	Aflasafe MWMZ01	81a	9b	10	85
	Aflasafe MZ02	87a	10b	3	67
	Non-treated fields	39b	40a	21	4
	Maize (*n* = 102)				
	Aflasafe MWMZ01	86a	3	11b	90
	Aflasafe MZ02	96a	2	2b	88
	Non-treated fields	32b	17	51a	8
R10	Groundnut (*n* = 18)				
	Aflasafe MWMZ01	96a	1	3b	86
	Aflasafe MZ02	97a	0	3b	86
	Non-treated fields	48b	12	40a	20
	Maize (*n* = 246)				
	Aflasafe MWMZ01	89a	0	11b	75
	Aflasafe MZ02	97a	0	3b	80
	Non-treated fields	40b	3	57a	33

^Z^ This study was conducted in AEZ R4 (midland; Manica province), R7 (mid- to highland; Nampula, Niassa, and Zambezia provinces), R8 (lowland; Nampula province), and R10 (highland; Zambezia and Tete provinces). ^Y^ L – A. flavus L-morphotype, S – fungi with S morphology, and P – *A. parasiticus*. Frequency of isolation of *A. tamarii* was negligible and excluded from the study. Atoxigenic – percent of all fungi that did not produce detectable quantities of aflatoxins. ^X^ Percentages within each AEZ followed by different letters are significantly (*P* = 0.05) different according to least significant difference (LSD) test.

## Discussion

The current study evaluated two aflatoxin biocontrol products containing native atoxigenic isolates of *A. flavus* for their abilities to limit aflatoxin contamination in maize and groundnut fields managed by smallholder farmers across Mozambique. We found high effectiveness in aflatoxin control by the two tested products with most of the treated crops containing aflatoxin content meeting the EU and US standards for crops intended for human consumption. The effectiveness was primarily driven by the dominance of atoxigenic isolates in treated soils and crops, which competitively displaced aflatoxin producers and prevented them from becoming associated with the treated crops. In contrast, high proportions of the non-treated maize and groundnut crops did not meet standards and this was associated with high percentages of toxigenic fungi. This is the first formal report of atoxigenic-based aflatoxin biocontrol technology in Southern Africa, a region that suffers from recurrent aflatoxin contamination events (Meijer et al., 2021). Members of all the VCGs of the isolates composing Aflasafe MWMZ01 are also native to Malawi (*unpublished*). The fungal community structures and molecular studies that led to the selection of the genotypes composing the two products will be reported in a separate publication.

In non-treated crops, the aflatoxin accumulation was greater in groundnut than in maize across all AEZs, and aflatoxin prevalence was highest in R8 followed by R7 AEZ. These agro-ecologies, R8 in particular, are typically hotter and experience dry spells (IIAM, 2006) during the last quarter of the cropping season which coincides with crop stages near physiological maturity. This combination of factors may result in drought stressed crops near harvesting, potentially catalyzing an increase in aflatoxin prevalence in vulnerable crops like groundnut and maize (Cole et al., 1984; Payne, 1998). The high aflatoxin prevalence in both groundnut and maize is likely to exacerbate in the future as the country has been experiencing a more erratic climate and dry and warm spells from El Niño events.

Both biocontrol products, namely Aflasafe MWMZ01 and Aflasafe MZ02, were highly effective in reducing aflatoxin accumulation in groundnut and maize. This effectiveness was particularly noticeable in AEZs R7 and R8 where the aflatoxin prevalence in non-treated crops was higher. Similar high protection of biocontrol in maize and groundnut in highly aflatoxin-prone areas has been documented (Abbas et al., 2006; Agbetiamegh et al., 2020). Most fields treated with biocontrol products conformed to EU and US regulations relating to maximum aflatoxin content allowed in food for human consumption.

The Aflasafe trademark with country-specific biocontrol products developed by IITA, USDA-ARS, and several partners has been widely tested, its effectiveness demonstrated, validated, and promoted in SSA in groundnut and maize (Bandyopadhyay et al., 2022; Ortega-Beltran and Bandyopadhyay, 2023). However, prior to the current study, reports for biocontrol effectiveness in Southern African countries were not available. The aflatoxin reduction in maize and groundnut in Mozambique following the application of biocontrol products, containing native atoxigenic isolates of *A. flavus* belonging to VCGs with wide distribution in the country, is in line with findings from other African countries where country-specific Aflasafe products were developed. In Kenya, between 80 and 96% of the maize from fields treated with a Kenyan version of Aflasafe, KE01, conformed to the EU regulations (Bandyopadhyay et al., 2016; Migwi et al., 2020). In Nigeria, the application of mixture of four endemic atoxigenic VCGs of the Nigerian Aflasafe™ reduced aflatoxin accumulation in maize by 67–95% (Atehnkeng et al., 2014). The genome of the four Nigerian Aflasafe VCGs in relation to their atoxigenicity and phylogenetic analysis has recently been characterized (Chang, 2022; Legan et al., 2024). Likewise, Aflasafe SN01 was effective in reducing aflatoxin accumulation in groundnut fields during 5 years by 58–98%, at harvest, and 76–96%, after storage, in Senegal (Senghor et al., 2020), and by up to 100% during 2 years in maize and groundnut fields at harvest in The Gambia (Senghor et al., 2021). In Tanzania, groundnut and maize fields treated with local biocontrol products Aflasafe TZ01 and Aflasafe TZ02 had aflatoxin reduction by 30–92% (Mahuku et al., 2023). Even greater aflatoxin reductions (99%) were reported in Ghana in maize and groundnut from fields treated with Aflasafe GH01 and Aflasafe GH02 (Agbetiamegh et al., 2020). Aflasafe products continue to be developed for several SSA countries using native atoxigenic *A. flavus* VCGs.

The reduction in aflatoxin accumulation in crops following biocontrol treatment at the right time is associated with founder effects (Ortega-Beltran and Cotty, 2018) and the displacement of native aflatoxigenic fungi by the adapted and highly competitive atoxigenic active ingredient isolates of *A. flavus*. This event is characterized by a shift in soil and crop *Aspergillus* community from a considerably toxigenic to a mostly atoxigenic one while maintaining the overall equilibrium of fungal population density (Cotty, 1994; Abbas et al., 2006; Atehnkeng et al., 2022). In a long-term persistence of a biocontrol VCG in treated maize fields in Italy, the densities of the toxigenic isolates, detected monitoring the *aflR* gene, were 45% in non-treated fields compared to 22% in the treated fields (Ouadhene et al., 2023). In other studies, displacement of toxigenic fungi by active ingredient fungi in biocontrol formulations was tracked with VCG analyses (Agbetiamegh et al., 2020; Atehnkeng et al., 2022). Yet other studies have assessed the displacement by evaluating aflatoxin-producing abilities of the fungi associated with treated and non-treated soils and crops (Weaver and Abbas, 2019). In the current study, we followed the latter approach. Aflatoxin-production potentials of all recovered isolates were evaluated and percentages of atoxigenic isolates were calculated. Although we did not evaluate which applied atoxigenic VCG the atoxigenic isolates belong to, the results showed a high proportion of atoxigenic isolates in treated soils and crops.

The prevalence of the aflatoxigenic species varies with crop and geographic location (Klich, 2007; Probst et al., 2014). Diener et al. (1987) and Horn and Dorner (1998) attributed economic importance to *A. parasiticus* only in groundnut and suggested that only rarely is isolated in other crops. In the present study as well as other studies in Mozambique (Augusto et al., 2014) and Zambia (Kachapulula et al., 2017) in Southern Africa, both *A. flavus* L-morphotype and *A. parasiticus* were the dominant fungi in soil, maize grain, and groundnut kernels from non-treated fields across locations and the bulk of these were toxigenic. The displacement of toxigenic isolates by the atoxigenic isolates of *A. flavus* in the soil occurred after the application of biocontrol products. Likewise, atoxigenic isolates of *A. flavus* were dominant in the maize grain and groundnut kernels from treated fields at harvest, and this dominance continued during storage. The high proportions of atoxigenic fungi in treated crops were consistently associated with lower aflatoxin concentrations. In contrast, higher aflatoxin content in non-treated crops was accompanied by high proportions of toxigenic fungi belonging to the three major types of fungi. Overall, results from the current study indicate that toxigenic fungi were displaced by the atoxigenic active ingredients in soils and harvested crops from treated fields with proportional aflatoxin reduction. Further studies are needed to exploit the longevity and mechanisms of the displacement under diverse environments and ascertain to which applied VCGs the recovered and dominant atoxigenic isolates belong to.

Results of laboratory studies on recombination between atoxigenic and toxigenic fungi were used to suggest such recombination if occurring in the field could result in progenies with altered aflatoxin producing abilities (Olarte et al., 2012) and results of a field study suggested recombination might occur in fields as early as 3 months after application of biocontrol products (Molo et al., 2022). The field study used population genetics tools to test the hypothesis that products containing isolates from certain lineages would result in higher chances of recombination, potentially leading to lower aflatoxin production potentials in fungi interacting with treated crops. That study claimed to demonstrate that aflatoxin producing potential of isolates from treated fields differ from those in non-treated fields. However, only 99 isolates were examined from the 3-year evaluation of multiple treatments in multiple states. Further, since aflatoxin content in the grain was not measured, it remains to be seen whether recombination would reduce aflatoxin in the crop during the year of treatment, which is the key goal of farmers using the technology. Previous studies have shown no evidence of gene flow between VCGs of *A. flavus* (Grubisha and Cotty, 2010, 2015). Indeed, no introgression of aflatoxin producing ability was detected in 237 isolates belonging to the same VCG of the longest used commercial atoxigenic active ingredient, AF36, which was also included in the study by Molo et al. (2022). The study by Grubisha and Cotty used vegetative compatibility analyses to identify isolates collected from environmental sources and treated soils over a 14- year period (Grubisha and Cotty, 2015).

All four isolates in Aflasafe MWMZ01 possess the *MAT1-2* mating-type, while Aflasafe MZ02 contains one isolate with *MAT1-1* and three with *MAT1-2* (*unpublished results*). In the current study, fungi in soils and crops at harvest from the 684 fields treated with either product were mostly atoxigenic regardless of mating-type profiles of the active ingredients in the applied products. In addition, the dominance of atoxigenic fungi was maintained in harvested crops after 3 months of storage. These findings are not in consonance with the deduction of Olarte et al. (2012) and the hypotheses proposed by Molo et al. (2022). Our group has consistently reported high frequencies of applied atoxigenic VCGs and low aflatoxin levels in crops treated with products containing multiple active ingredient fungi (Bandyopadhyay et al., 2022; Senghor et al., 2020, 2021; Agbetiamegh et al., 2020; Mahuku et al., 2023), even up to 3 years after application (Atehnkeng et al., 2022). Results across multiple countries provide evidence that treating crops with biocontrol is effective, primarily due to restructuring of *Aspergillus* communities so that the applied atoxigenic VCGs become a larger proportion. The findings of the current study, along with those of our previous studies, support competitive displacement, and not recombination of specific isolates of different mating-types, as the primary mechanism for aflatoxin biocontrol. The data and conclusions reported by Ouadhene et al. (2024) further reinforce this inference.

The current study did not attempt to evaluate the persistence of the atoxigenic isolates of *A. flavus* used in the two evaluated biocontrol products, but when products contain native atoxigenic isolates, these are capable of persisting for long-term in treated agro-ecosystems (Atehnkeng et al., 2022; Ouadhene et al., 2023) and they offer non-target benefits without being detrimental to other soil resident microflora (Bhandari et al., 2020; Zhu et al., 2022).

Aflatoxin tolerance thresholds may change over time, or new ones could be established in countries that currently lack them. Should these changes occur, the categorization of thresholds into ≤4 ppb, ≤20 ppb, and > 20 ppb might require adjustments. In the current study, the categorization was done to show how many groundnut and maize samples from treated or untreated fields fell within common ranges across countries and to show biocontrol effectiveness. The full scope of aflatoxin levels, without categorization, across various treatments and agro-ecologies is shown in Figure 3.

A combination of pre- and post-harvest aflatoxin management practices is advocated and encouraged and biocontrol with native atoxigenic isolates of *A. flavus* belonging to widely distributed atoxigenic VCGs is a key component of this package. Ultimately, eliminating or significantly reducing the burden of aflatoxin contamination in food crops will go a long way toward improving health of the rural poor, enhancing food nutritional intake particularly in children and, therefore, reduce stunting, and increasing income generation of groundnut and maize farmers through access of premium markets. This study offers first hard evidence of efficacious and accessible aflatoxin management tool to maize and groundnut farmers in Mozambique. Now, after the registration of the two biocontrol products with regulatory authorities in Mozambique, IITA has licensed manufacturing and distribution responsibilities of aflatoxin biocontrol to AflaLivre®, a private company in Mozambique that works with 6,000 smallholder farmers but with a horizon to expand. With its new manufacturing facility in Nampula, the company has the capacity to produce biocontrol for use in Mozambique, and neighboring countries for which technology is approved by national regulators for commercial use (i.e., Malawi and Zambia) or in the process of developing it (i.e., Madagascar).

## Conclusion and recommendations

This study identifies aflatoxin hot-spots for maize and groundnut in central and northern Mozambique on the north–south axis and from the coastal lowlands to the interior midlands on the east–west axis. These agro-ecologies, specifically R8 and R7, experience semi-arid to sub-humid climates with annual rainfall of 800 to 1,200 mm (IIAM, 2006) and are prone to dry spells and high temperatures during crop stages vulnerable to increasing aflatoxin risk. Maize and groundnut grown in R7 and R8 are more vulnerable to aflatoxin contamination than highland areas with cooler, more humid climates (Augusto et al., 2014). The two biocontrol products evaluated in the current study each contains four distinct atoxigenic *Aspergillus* strains native to Mozambique. Both products are very effective at reducing aflatoxin formation in both groundnut and maize by displacing toxigenic *Aspergillus* in treated soil. Aflatoxin reductions were more pronounced in aflatoxin hot-spot areas than in highland regions. This is significant because current aflatoxin management practices rely primarily on cultural crop management, marginal varietal resistance, and sound post-harvest practices that are insufficient when toxigenic *Aspergillus* predominate in soils. Integration of Aflasafe biocontrol products with other mitigation practices offers a sustainable mid- to long-term solution to reduce toxigenic inoculum buildup and aflatoxin accumulation in crops.

The current work suggests safe levels of aflatoxins in maize and groundnut in Mozambique are achievable with integrated management incorporating Aflasafe. Management needs to extend along the entire value chain in partnership with key stakeholders. The commercial-scale manufacturing and distribution of biocontrol products by the licensed company, AflaLivre, are essential prerequisites for scaling up. Economic incentives for commercialization may result from the enforcement of legal limits for aflatoxins in food and feed. Currently, groundnut and maize enter formal and informal market channels without adequate consideration of aflatoxins. Understandably due to high poverty levels, stakeholders across value chains, including farmers, off-takers, retailers, traders, and consumers, tend to prioritize quantity and superficial quality over food safety. Therefore, it is necessary to create continuous and consequential awareness among relevant Government institutions, lawmakers, and public and private players in the maize and groundnut value chains about aflatoxin hazards and benefits of biocontrol.

Lastly, donor funding, partnerships, and collaborations are key to scaling up the use of atoxigenic strain-based biocontrol. These resources will support the refinement of biocontrol formulations and application methods and timing to fit the needs of smallholder farmers. Widespread training to inform smallholders and address concerns about biocontrol will also benefit adoption.

## Data Availability

The raw data supporting the conclusions of this article will be made available by the authors, without undue reservation.
